# CXCL4/Platelet Factor 4 is an agonist of CCR1 and drives human monocyte migration

**DOI:** 10.1038/s41598-018-27710-9

**Published:** 2018-06-21

**Authors:** James M. Fox, Fahima Kausar, Amy Day, Michael Osborne, Khansa Hussain, Anja Mueller, Jessica Lin, Tomoko Tsuchiya, Shiro Kanegasaki, James E. Pease

**Affiliations:** 1 Receptor Biology Group, Inflammation, Repair and Development Section, National Heart and Lung Institute, Faculty of Medicine, Imperial College London, London, SW7 2AZ UK; 2 Research Institute, National Center for Global Health and Medicine, 1-21-2 Toyama, Shinjuku-ku, Tokyo, 162-8655 Japan; 3 Present Address: Department of Biology, University of York, Heslington, York YO10 5DD UK; 4 Present Address: School of Pharmacy, UEA, Norwich NR4 7TJ UK

## Abstract

Activated platelets release micromolar concentrations of the chemokine CXCL4/Platelet Factor-4. Deposition of CXCL4 onto the vascular endothelium is involved in atherosclerosis, facilitating monocyte arrest and recruitment by an as yet, unidentified receptor. Here, we demonstrate that CXCL4 drives chemotaxis of the monocytic cell line THP-1. Migration and intracellular calcium responses induced by CXCL4 were *pertussis* toxin-sensitive, implicating a GPCR in signal transduction. Cell treatment with chondroitinase ABC ablated migration, suggesting that *cis* presentation of CXCL4 by cell surface glycosaminoglycans to a GPCR is required. Although CXCR3 has been previously described as a CXCL4 receptor, THP-1 cells were unresponsive to CXCR3 ligands and CXCL4-induced migration was insensitive to a CXCR3 antagonist, suggesting that an alternative receptor is involved. Interrogating CC-class chemokine receptor transfectants, we unexpectedly found that CXCL4 could induce the migration of CCR1-expressing cells and also induce CCR1 endocytosis. Extending our findings to primary human monocytes, we observed that CXCL4 induced CCR1 endocytosis and could induce monocyte chemotaxis in a CCR1 antagonist-sensitive manner. Collectively, our data identify CCR1 as a previously elusive monocyte CXCL4 receptor and suggest that CCR1 may play a role in inflammation where the release of CXCL4 is implicated.

## Introduction

Chemokines represent a large family of small peptides that typically signal via G protein-coupled receptors (GPCRs) and which recruit leukocytes to inflammatory sites and lymphoid microenvironments^1^. Chemokines are classified as belonging to one of four distinct groups based on the conservation of amino-terminal cysteine residues^2^. The chemokines belonging to two main classes contain a pair of cysteine residues, which are either adjacent (CC class) or separated by a single amino acid (CXC class). Signaling is considered to be class-restricted, with CC-chemokines activating CC receptors and CXC chemokines activating CXC receptors. Both CC and CXC chemokines have been shown to be highly expressed in the atherosclerotic plaques of humans and rodents, implying that enhanced leukocyte recruitment to the plaque is a driver of disease^3^. Supportive of this, deletion of several key chemokine receptors has been shown to protect against the development of atherosclerosis in murine models^4–6^, suggesting that targeting chemokine receptors may be therapeutically beneficial^7^.

CXCL4/Platelet Factor-4 was the first member of the chemokine family to be discovered^8^ and is found at significant levels in atherosclerotic plaques, where its abundance correlates with lesion severity^9^. Several lines of evidence suggest that CXCL4 is an important player in atherogenesis. CXCL4 is released in micromolar concentrations following platelet activation and its deposition on the endothelium has been shown to exacerbate atherosclerotic lesion formation in Apolipoprotein E-deficient mice^10^. Consistent with this role, deletion of CXCL4 on the same genetic background results in reduced atherosclerotic lesion size^11^. CXCL4 exerts numerous effects on monocytes, although its roles appear to be complex. There is some debate as to whether or not CXCL4 alone is able to induce monocyte migration^12,13^, although CXCL4 has been shown to form functional heterodimers with CC chemokines such as CCL5/RANTES that promote monocyte arrest on endothelium^14^. These CXCL4/CCL5 heterodimers are atherogenic, since inhibition of their formation by small molecule antagonists is protective in a mouse model of atherosclerosis^15^.

Although the chemokine receptors CXCR3-A and CXCR3-B have been shown to mediate endothelial and T-cell signaling in response to CXCL4^16–18^, the receptor by which CXCL4 signals in human monocytes has proved elusive. This has hampered efforts to further elucidate the biology of this chemokine. In this study we provide several clear lines of evidence to demonstrate that CXCL4 is an agonist of the CC chemokine receptor CCR1 and that this receptor mediates CXCL4-signaling in human monocytes. This has implications for its targeting in the treatment of atherosclerosis and other inflammatory diseases.

## Results

### Functional responses induced by CXCL4 treatment of monocytic THP-1 cells

**Figure 1:**
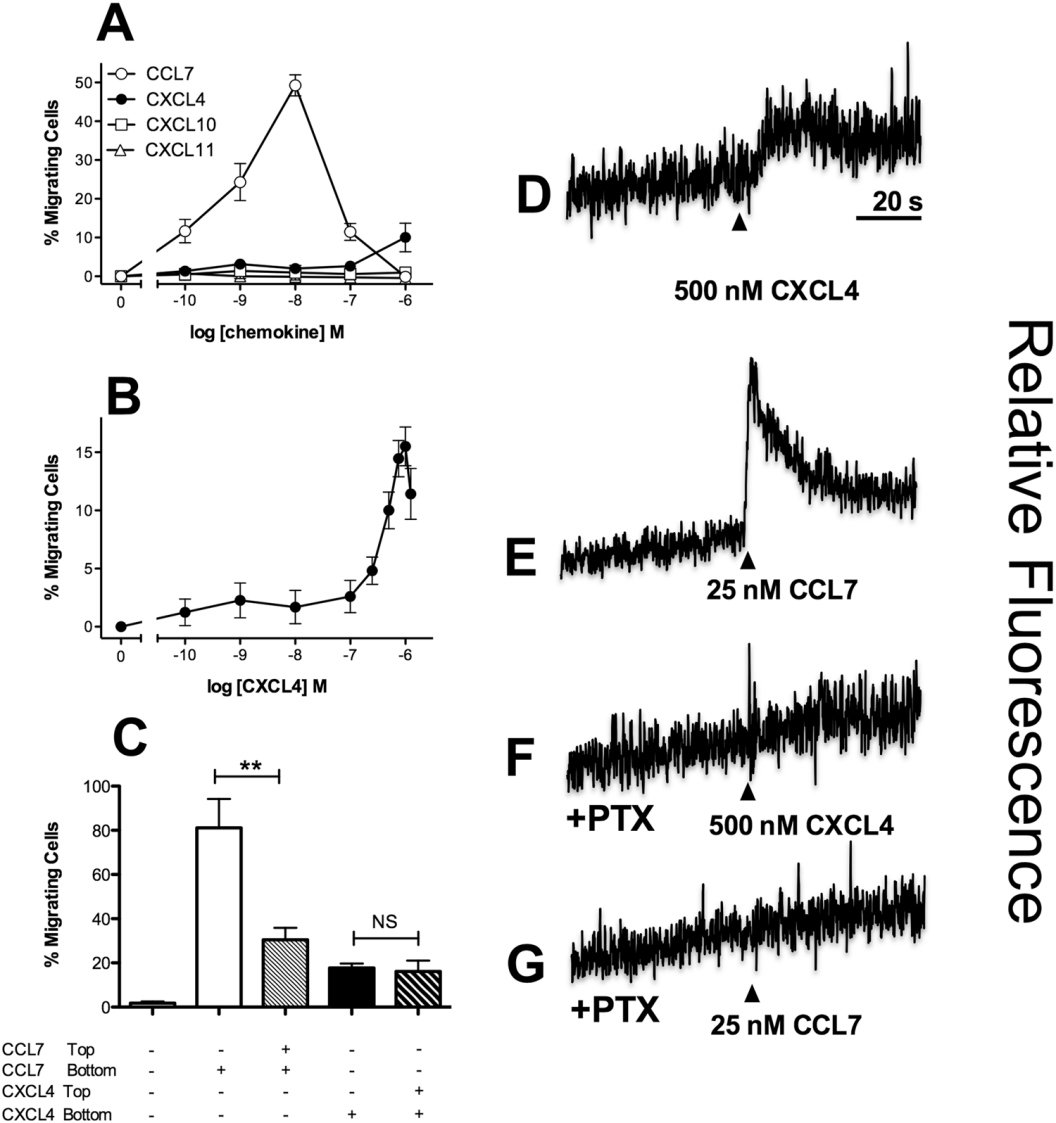
THP-1 cells respond to CXCL4 - Panels A and B. Migratory responses of THP-1 cells to increasing concentrations of CXCL4, CCL7, CXCL10 and CXCL11 (n = 4, Panel A; n = 3 Panel B). Panel C. Migratory responses of THP-1 cells to 1 *μ*M CXCL4 or 10 nM CCL7 in the presence or absence of a concentration gradient (n = 4). Panels D-G. Intracellular calcium flux of THP-1 cells in response to CXCL4 and CCL7, following culture in the absence (Panels D, E) and presence (Panels F, G) of *pertussis* toxin (PTX). Arrowheads indicate point of chemokine addition (representative of n = 3).

The monocytic line THP-1 was initially examined by chemotaxis assays using a modified Boyden chamber. Cells were highly responsive to CCL7, as we have previously described^19^, with a 10 nM concentration of CCL7 inducing a robust response (Fig. 1A). Migration to CXCL4 was less potent, with migration only observed at a concentration of 1 *μ*M CXCL4. No migration of THP-1 cells to the CXCR3 ligands CXCL10 and CXCL11 was observed (Fig. 1A), nor could we detect significant levels of cell surface CXCR3 expression with a specific antibody nor specific binding of radiolabeled CXCL10 (Supplementary Fig. 1). This suggests that in contrast to CXCL4 signaling in T cells^17,18^, CXCL4 responses in THP-1 cells are not mediated by either of the CXCR3 variants. This was supported by the lack of effect of a CXCR3 antagonist on CXCL4 induced responses (Supplementary Figure 1) and is in agreement with other studies citing either a lack of CXCR3-B mRNA in monocytes^20^ or no effect of a CXCR3 antagonist on CXCL4-induced monocyte responses^21^. Using an extended concentration range of CXCL4, a bell-shaped migratory curve typical of other chemokines was observed (Fig. 1B). In keeping with the findings of several groups studying CXCL4 signaling in a variety of cell types^12,22–24^, micromolar concentrations of CXCL4 were required to induce migration, suggesting that the affinity of CXCL4 for its receptor is likely to be low. To assess the relative contributions of chemokinesis and chemotaxis to THP-1 cell migration, optimal concentrations of CCL7 and CXCL4 were placed either in the bottom compartment of a modified Boyden chamber or in both the top and bottom compartments (Fig. 1C). Whilst the inclusion of CCL7 in the upper compartment significantly reduced THP-1 migration (indicative of a largely chemotactic component), the inclusion of CXCL4 in the upper compartment had no significant effect upon CXCL4-induced migration. This suggests that a gradient of CXCL4 is not required to induce migration of THP-1 cells when assayed with a modified Boyden chamber.

**Figure 2:**
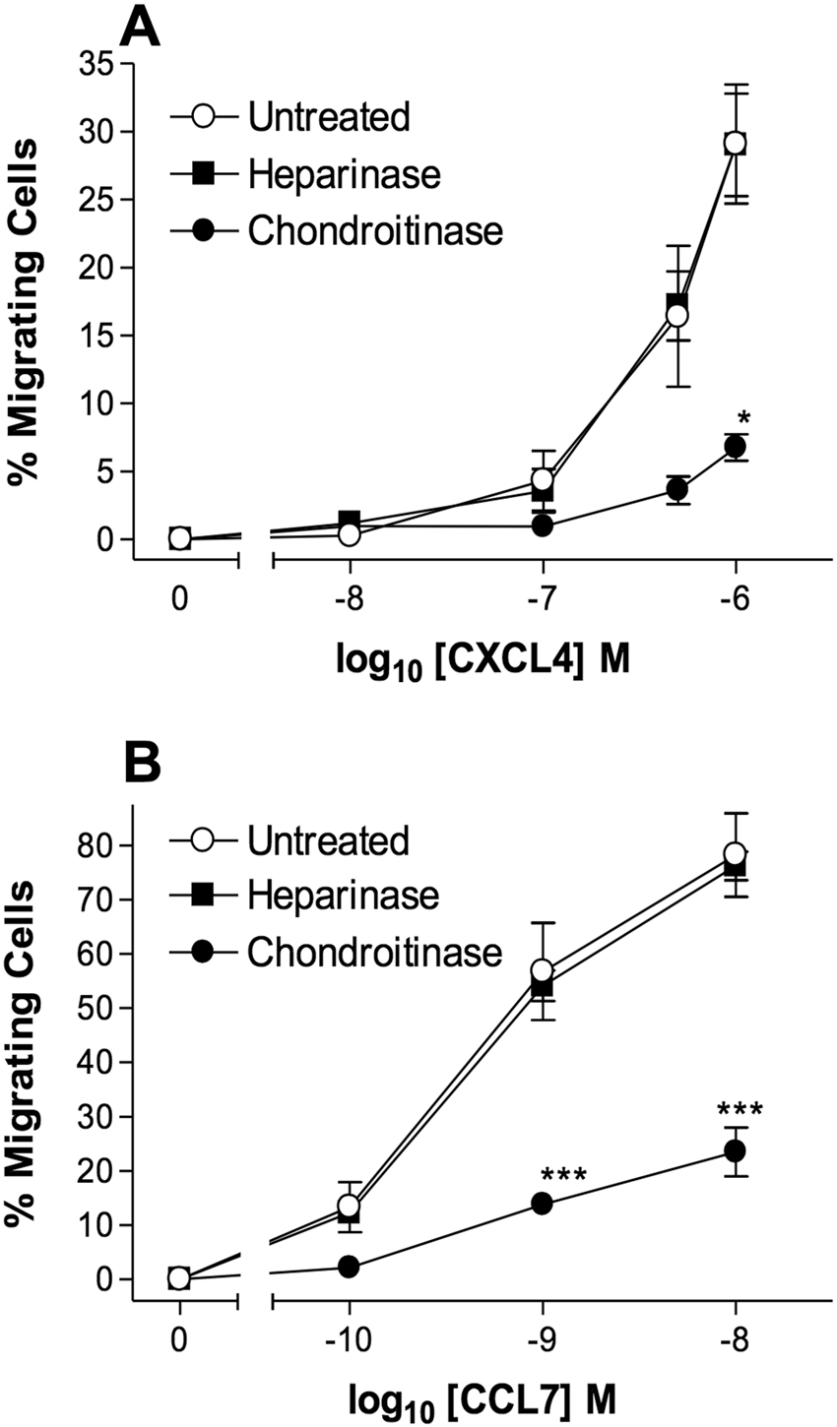
Chondroitin sulphates are required for chemotaxis - Chemotaxis of THP-1 cells in response to CXCL4 (Panel A) and CCL7 (Panel B), following incubation of cells in the presence and absence of heparinase and chondroitinase (n = 3).

CXCL4 treatment of THP-1 cells was also observed to give rise to an increase in intracellular calcium (Fig. 1D), as was treatment with the chemokine CCL7 (Fig. 1E). Pre-treatment of THP-1 cells with *pertussis* toxin ablated both the induction of intracellular calcium release (Fig. 1F and G) and THP-1 cell migration (Supplementary Fig. 1) in response to either CXCL4 or CCL7, suggesting that these signals are mediated via GPCRs that couple to G*α*i proteins. Petersen and colleagues have previously reported that CXCL4 binds to one or more chondroitin sulphate-decorated glycoproteins on the surface of neutrophils, which is necessary for the biological activity of CXCL4, since enzymatic removal of cell surface chondroitin ablates functional responses^25,26^. Similarly, we observed that pre-treatment of THP-1 cells with the enzyme chondroitinase ABC significantly reduced the migration of THP-1 cells to not only CXCL4 but also to CCL7, whilst heparinase treatment had no effect (Fig. 2A and B). These data suggest that one or more chondroitin sulphate-decorated glycoproteins present CXCL4 in *cis* to a specific GPCR.

**Figure 3:**
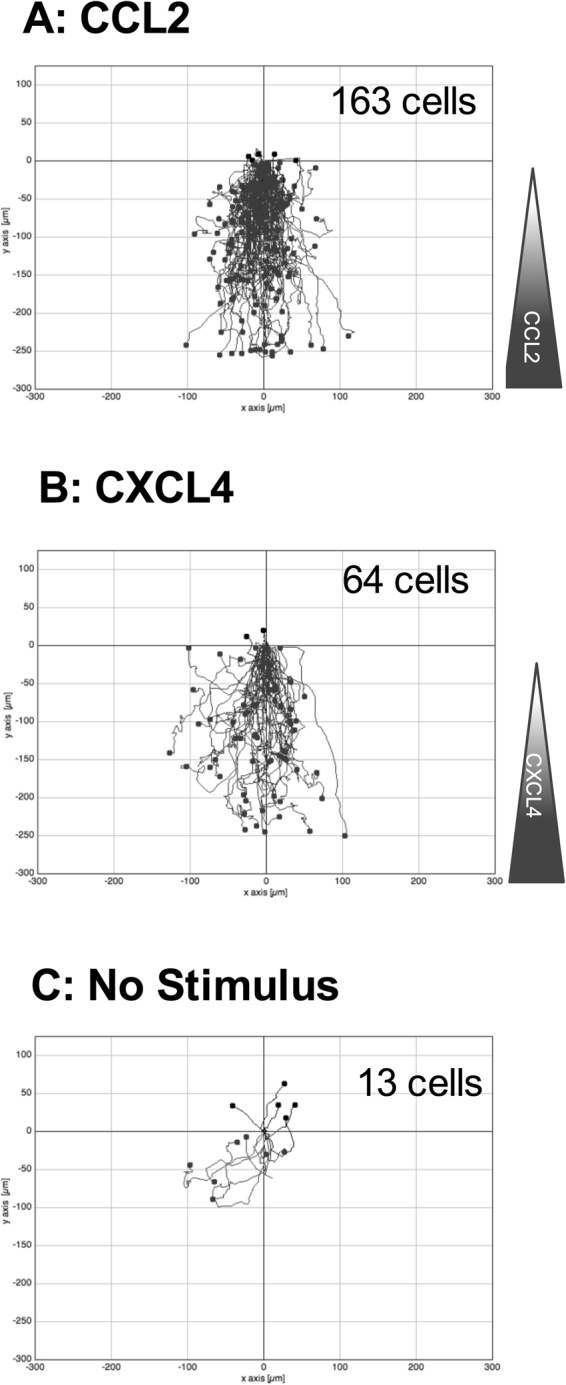
CXCL4 drives the chemotaxis of THP-1 cells as visualized via TAXIScan - Panels A-C show chemotaxis of THP-1 cells along a gradient of CCL2 (**A**), CXCL4 (**B**) as illustrated or in the absence of stimulation (**C**) as assessed for 2 hr at 37 °C. Data shown are pooled from replicate conditions from 3 individual experiments.

**Figure 4:**
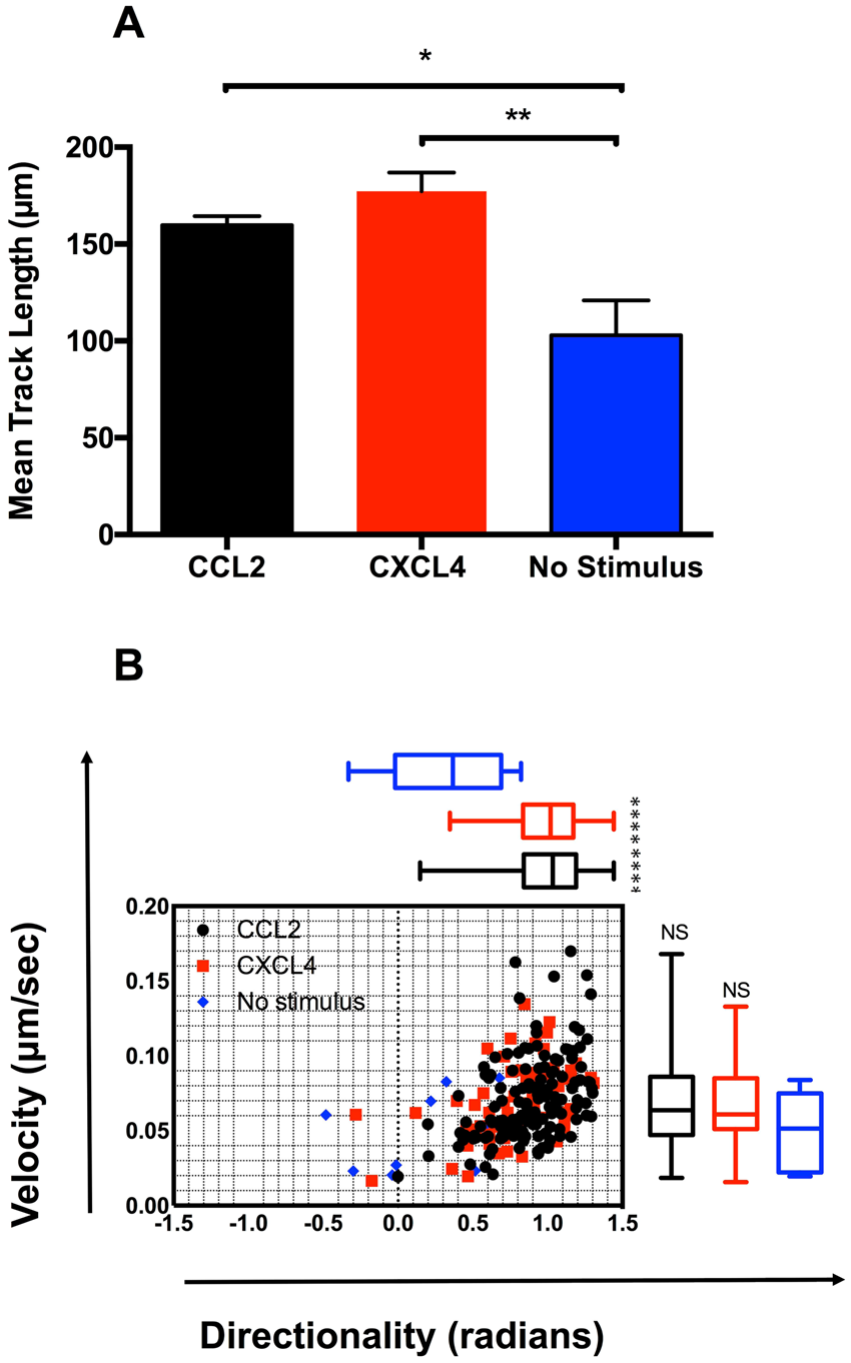
Analysis of THP-1 cell chemotaxis via TAXIScan - Panel A shows the mean track length observed for THP-1 cells exposed to the various stimuli using the data shown in Fig. 3. Panel B shows a Velocity-Directionality Box (VD-B) plot of the same data. Each symbol corresponds to an individual cell exposed to stimuli as indicated by the key. Median values of directionality and velocity (with the range) are are shown alongside as a box plot.

To supplement our Boyden chamber chemotaxis data, we switched to a novel horizontal device for imaging leukocyte migration in real time, namely the TAXIScan^27^. In this device, cells are introduced at one end of a 5 *μ*m deep microchannel and chemoattractant is introduced at the other. Migration of cells to chemoattractant within the channel is then monitored via a charge coupled device camera, with the data subjected to manual cell tracking (Supplementary Fig. 2). THP-1 cells were introduced into the chambers and their migration in response to CCL2, to CXCL4 or in the absence of stimulation was recorded over a 2 hr period (Fig. 3). Migration to CCL2 was typically robust, with many cells migrating towards the source of chemokine (Fig. 3A, Supplementary Video 1). Likewise, several cells were seen to migrate along a gradient of CXCL4 (Fig. 3B, Supplementary Video 2). In both cases, cells were seen to generate relatively long protrusions in the direction of the gradient prior to migration in the same direction. In contrast, relatively few cells migrated along the microchannel in the absence of stimulation (Fig. 3C, Supplementary Video 3). The tracks of individual cells were subjected to further analysis. The mean track length of cells responding to CXCL4 or CCL2 was seen to be significantly greater than that of cells not subjected to a stimulus (Fig. 4A). Velocity Directionality Box (VD-Box) plots of migration were subsequently generated, in which each symbol corresponds to an individual cell (Fig. 4B). In a visual system, better designed to assess cellular migration and differentiate between chemotaxis and chemokinesis, cells exposed to gradients of either CXCL4 or CCL2 had a significantly higher directionality value compared to unstimulated cells (plotted to the right most area of the graph) indicating that both CXCL4 and CCL2 induce the directional migration (chemotaxis) of THP-1 cells. The velocity of cells migrating in response to CXCL4 or CCL2 trended towards an increase over basal cell velocity but this was not significant.

### CXCL4 is an agonist of CCR1

**Figure 5:**
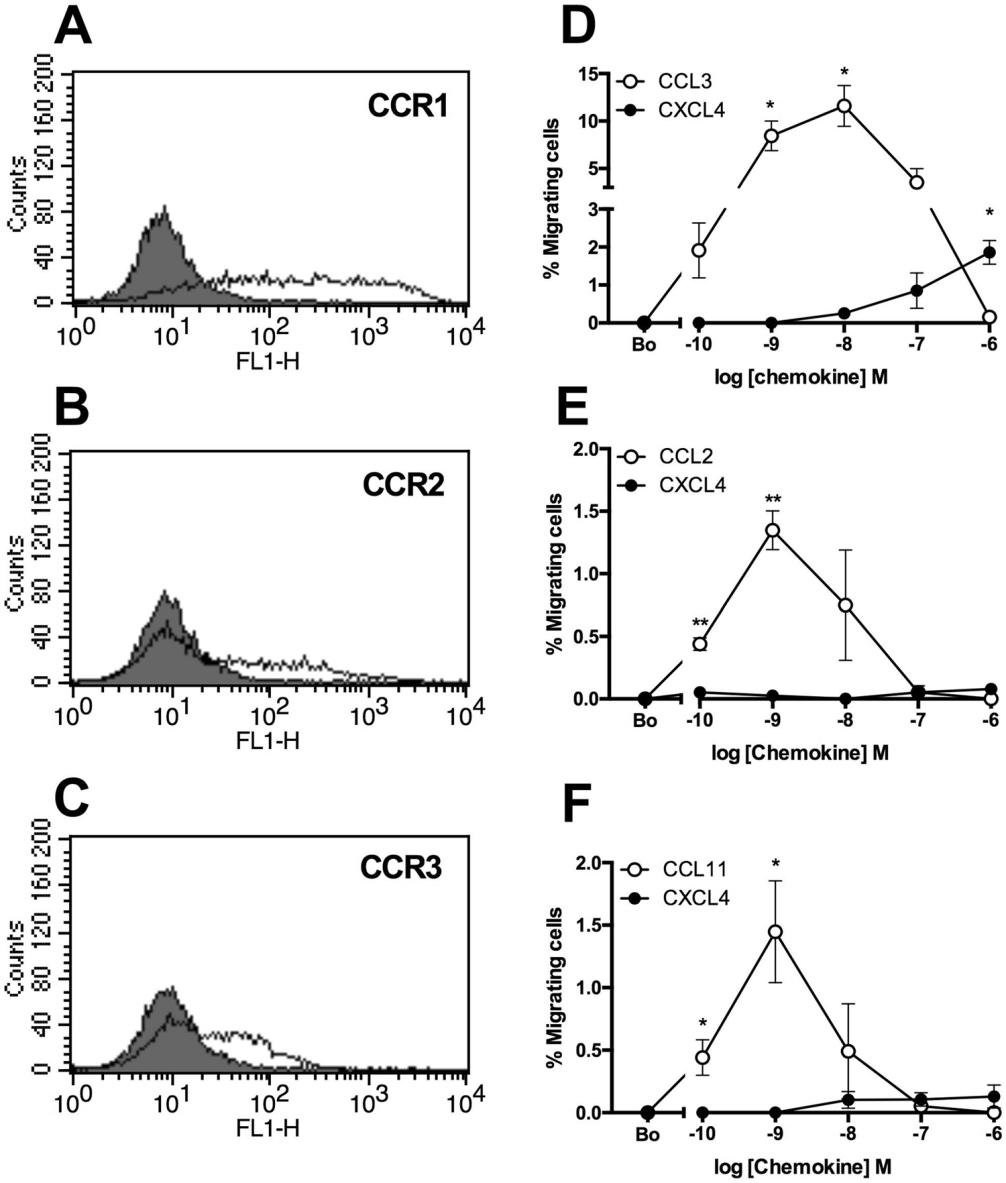
CCR1 mediates CXCL4-induced leukocyte migration in a transfectant system- Panels A, B, C show transient expression of HA-tagged variants of the known signaling CCL7 receptors, CCR1, CCR2 and CCR3 as assessed by flow cytometry using an HA-specific mAb (open histogram) or isotype control (filled histogram). Panels D, E, F show the migratory responses of the same transfectants to CXCL4 and to control chemokines (n = 3).

We have previously shown that responses to CCL7 in THP-1 cells are mediated predominantly via CCR1^19^. We observed that the most robust responses in the Boyden chamber assay to CXCL4 were obtained with THP-1 cells that were also the most responsive to CCL7. We therefore postulated that contrary to the current dogma that chemokine receptor activation is class-restricted (i.e. CXC chemokines activate only CXC receptors), CXCL4 might be acting by a known CCL7 receptor. To test this hypothesis, we transfected the mouse pre-B cell line L1.2 with plasmids encoding the three CC chemokine receptors at which CCL7 is known to be an agonist, namely CCR1, CCR2 and CCR3. All three receptors were readily expressed in a transient manner (Fig. 5A,B,C) and cells correspondingly responded to their cognate ligands CCL3, CCL2 and CCL11 in Boyden chamber assays (Fig. 5D,E,F). Notably, a significant response over basal migration to micromolar concentrations of CXCL4 was singularly observed in CCR1 transfectants (Fig. 5D), albeit a less potent and efficacious response than that observed with CCL3, suggesting that CXCL4 is a partial agonist of CCR1 in this assay.

**Figure 6:**
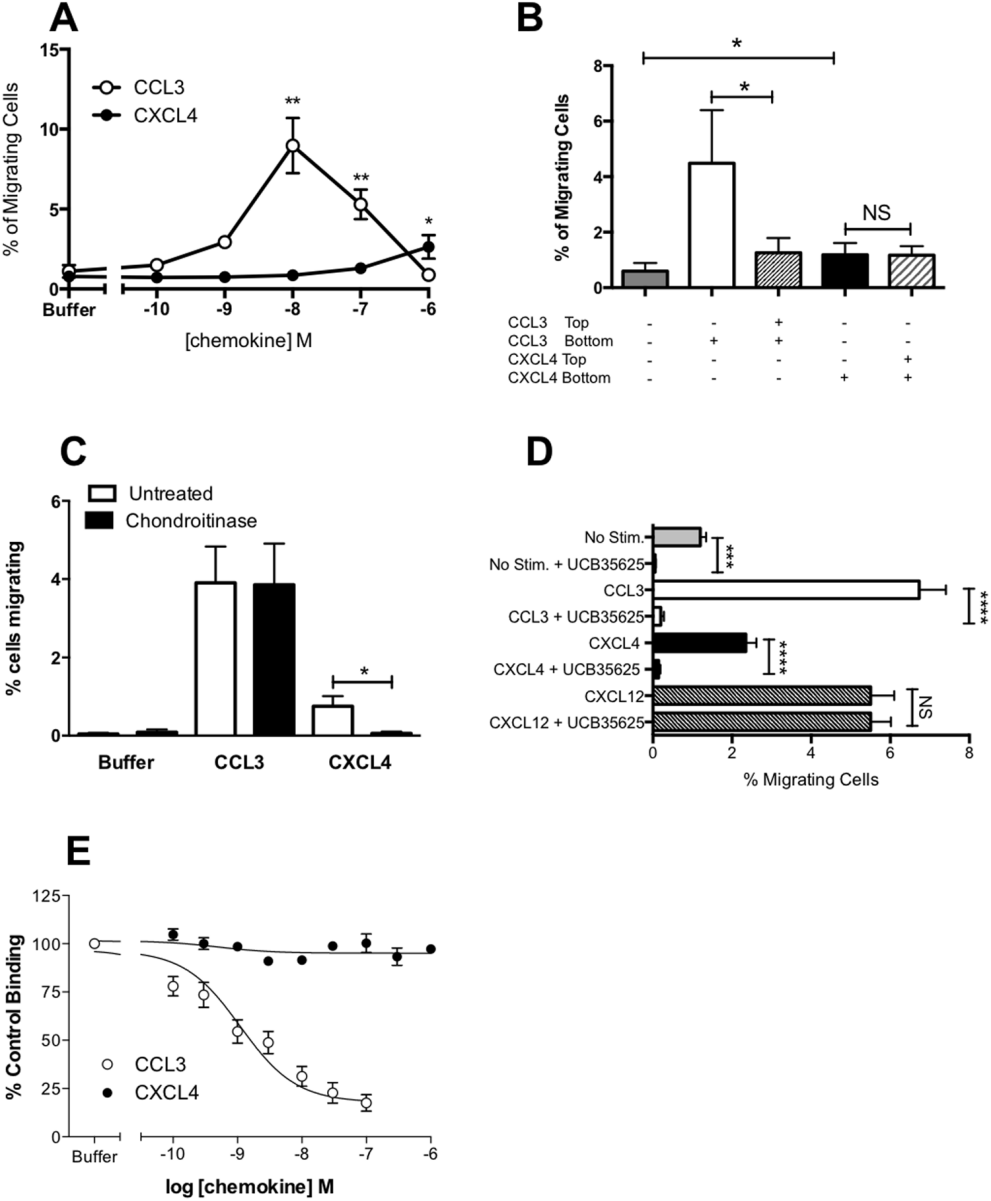
CXCL4 is an agonist of CCR1 - Panel A shows chemotactic responses to CCL3 and CXCL4 of an L1.2 transfectant line stably expressing CCR1 (L1.2-CCR1), (n = 6). Panel B shows migration of L1.2-CCR1 cells in a modified Boyden chamber to 1 *μ*M CXCL4 or 10 nM CCL3 in the presence or absence of a chemokine gradient (n = 4). Panel C shows migration of the L1.2-CCR1 cell line to the same stimuli following incubation of cells in the presence and absence of heparinase and chondroitinase (n = 4). Panel D shows the blockade of no stimulus, CCL3 and CXCL4 migratory responses in the same stable cell line by the CCR1-specific antagonist UCB 35625 (n = 7); responses to CXCL12 were unaffected (n = 4). Panel E shows competitive binding assays where 125I-CCL3 was displaced from L1.2-CCR1 cells with increasing concentrations of unlabeled CCL3 but not with CXCL4 (n = 4). Statistical values indicated refer to paired t-tests.

We next sought to validate whether a well-characterized small molecule antagonist of CCR1 named UCB 35625^28,29^ was able to block CXCL4 signaling. Using L1.2 cells stably expressing high levels of CCR1, we again observed robust migration responses in Boyden chamber assays to both CCL3 and CXCL4 (Fig. 6A). As we had observed with THP-1 cells, migration to CXCL4 in the Boyden chamber assay was unaffected by the inclusion of CXCL4 both above and below the filter (Fig. 6B). This was in contrast to CCL3 responses which were significantly inhibited by including CCL3 both above and below the filter. As was the case with THP-1 cells, (Fig. 2) preincubation of L1.2 cells expressing CCR1 with chondroitinase was found to significantly inhibit the migration to CXCL4 whilst responses to CCL3 remained intact (Fig. 6C). Preincubation of cells with a 10 *μ*M concentration of UCB 35625 was able to ablate migration to optimal concentrations of both CCL3 and CXCL4 but had no significant effect upon migration to the CXCR4 ligand CXCL12 (Fig. 6D). Unlike naïve L1.2 cells^29^, the L1.2 CCR1 transfectants displayed a detectable level of migration in the absence of chemokine but this was significantly increased by exposure to 1 *μ*M CXCL4 (p = 0.0025). Basal migration was significantly inhibited by preincubation with UCB 35625, suggesting that when expressed at high levels, CCR1 shows a degree of constitutive activity, which is able to drive migration, as previously reported by Gilliland and colleagues^30^. Ligand binding assays were also undertaken in which CXCL4 was observed to be unable to compete with radiolabelled CCL3 for binding to CCR1, unlike unlabeled CCL3 which was able to displace radiolabelled CCL3 with ease (Fig. 6E).

**Figure 7:**
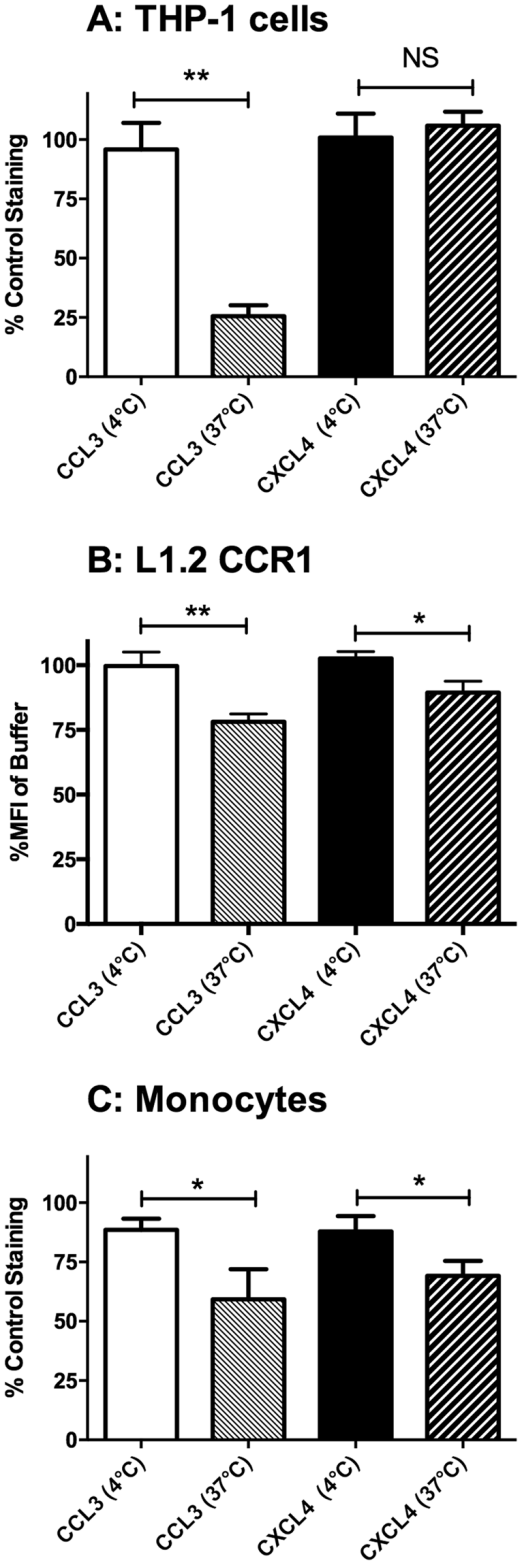
Induction of CCR1 endocytosis by CXCL4. - Panels A, B and C show CCR1 endocytosis in THP-1 cells (**A**) L1.2-CCR1 cell line (**B**) and freshly isolated human monocytes (**C**) following incubation with CCL3 or CXCL4 at 37 °C or 4 °C Data are from 4, 5 and 5 experiments respectively. Statistical values indicated refer to paired t-tests.

In addition to initiating receptor signaling, chemokines also classically induce receptor endocytosis following binding. We therefore assessed the ability of 50 nM CCL3 or 1 *μ*M CXCL4 to induce receptor endocytosis in THP-1 cells, the L1.2-CCR1 line and freshly isolated human monocytes (Fig. 7). Incubation of THP-1 cells with CCL3 at 37 °C induced a significant loss of cell surface CCR1 whilst CXCL4 was without significant effect (Fig. 7A). In contrast, when L1.2 CCR1 transfectants were examined, incubation with either CCL3 or CXCL4 was able to induce significant CCR1 endocytosis (Fig. 7B). Similarly, when human monocytes were examined, both CCL3 and CXCL4 induced significant CCR1 endocytosis at levels similar to those induced in the L1.2 CCR1 cell line. Collectively, these data confirm our findings that CXCL4 is a CCR1 agonist, although the cellular background in which CCR1 is expressed appears to dictate the efficacy of CXCL4-induced endocytosis.

### Blockade of CCR1 ablates monocyte migration in response to CXCL4

**Figure 8:**
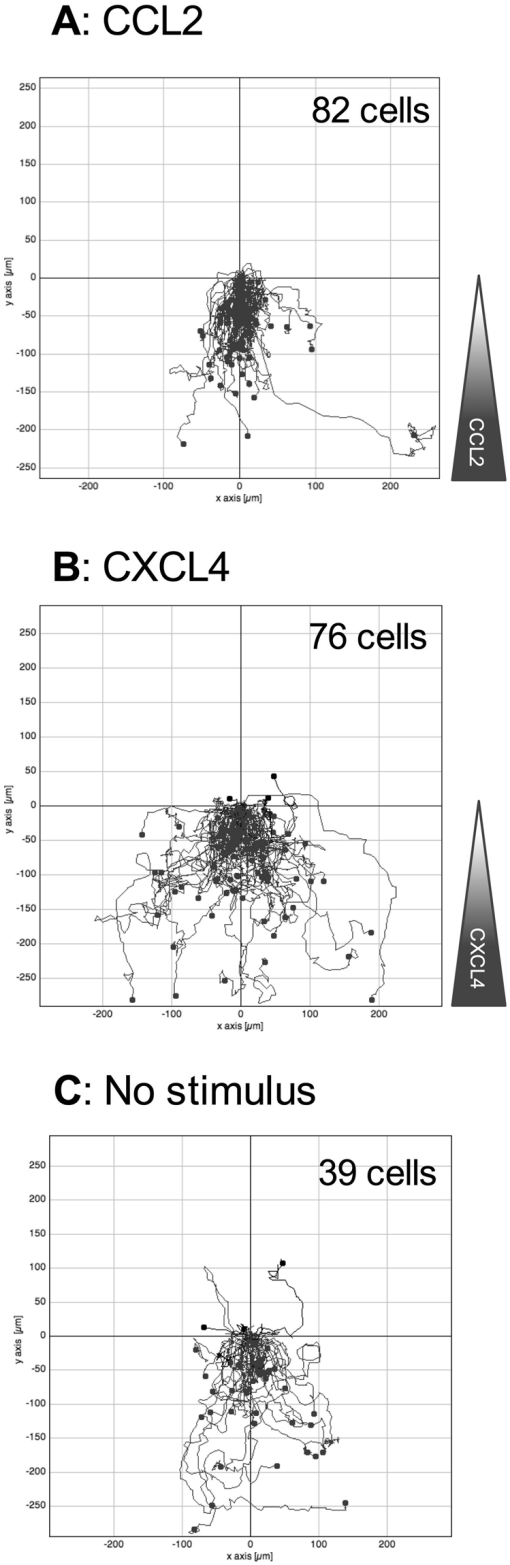
CXCL4 drives the chemotaxis of human monocytes. Panels A–C show migration of monocytes along gradients of CCL2 (**A**) or CXCL4 (**B**) compared to no stimulus (**C**) as assessed by TAXIScan for 2 hr at 37 °C. Data shown are pooled from replicate conditions (n = 6). The total number of cells migrated is shown in the upper right hand corner.

**Figure 9:**
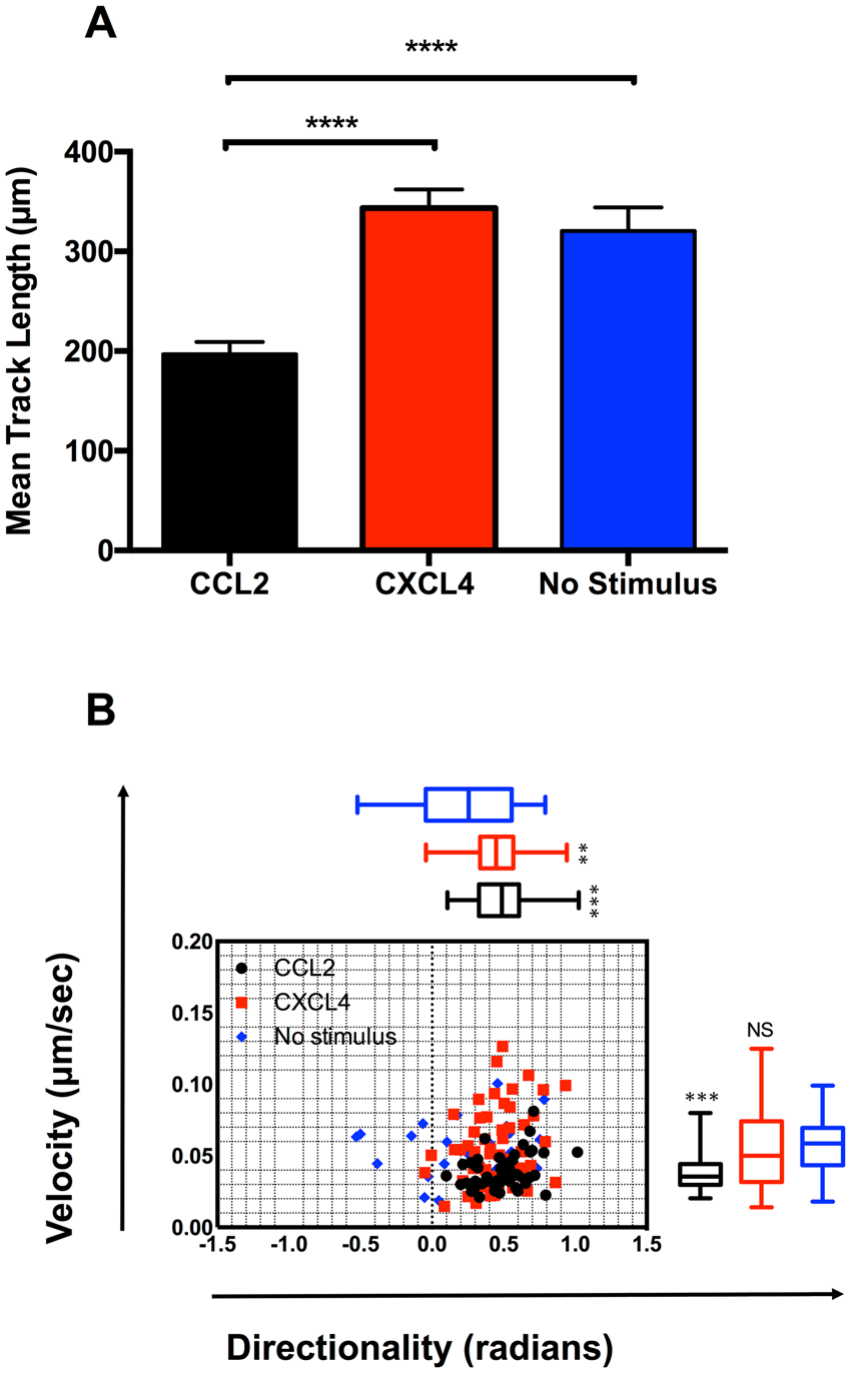
Analysis of monocyte chemotaxis via TAXIScan - Panel A shows the mean track length traveled by cells in response to each stimulus using the data shown in Fig. 8. Panel B shows a Velocity-Directionality Box (VD-B) plot of the same data. Each symbol corresponds to an individual cell exposed to stimuli as indicated by the key. Median values of directionality and velocity (with the range) are shown alongside as a box plot.

Having implicated CCR1 in CXCL4 signaling using a transfectant system and a monocytic cell line we sought to translate our migratory findings to assays using freshly isolated human monocytes. The TAXIScan device^27^ was employed to examine realtime migration of monocytes. This system also circumvents the problems of monocytes adhering to the filters of modified Boyden chambers, which can make analysis problematic. Exposure of monocytes to a gradient of CCL2 and CXCL4 resulted in robust migration, whilst considerably fewer monocytes were observed to migrate in the absence of chemokine (Fig. 8A,B,C, Supplementary Videos 4, 5 and 6). Unlike the THP-1 cells, no long protrusions were observed to be generated prior to migration. Tracking of individual monocytes revealed that the mean track length of monocytes migrating along a gradient of CCL2 was significantly shorter than those of monocytes exposed to a gradient of CXCL4 or not exposed to a stimulus (Fig. 9A). This finding was further clarified when the directionality and velocity parameters were assessed in a VD-B plot (Fig. 9B). Cells migrating in response to both CCL2 and CXCL4 had significantly greater directionality than those undergoing basal migration. In contrast, when the velocity of migration was examined, CCL2 stimulation resulted in significantly slower migration than observed in either basal migration or migration in response to CXCL4 stimulation. Collectively, these data show that that both CCL2 and CXCL4 induce the chemotaxis of monocytes, with CCL2 inducing a slower, more methodical migration.

**Figure 10:**
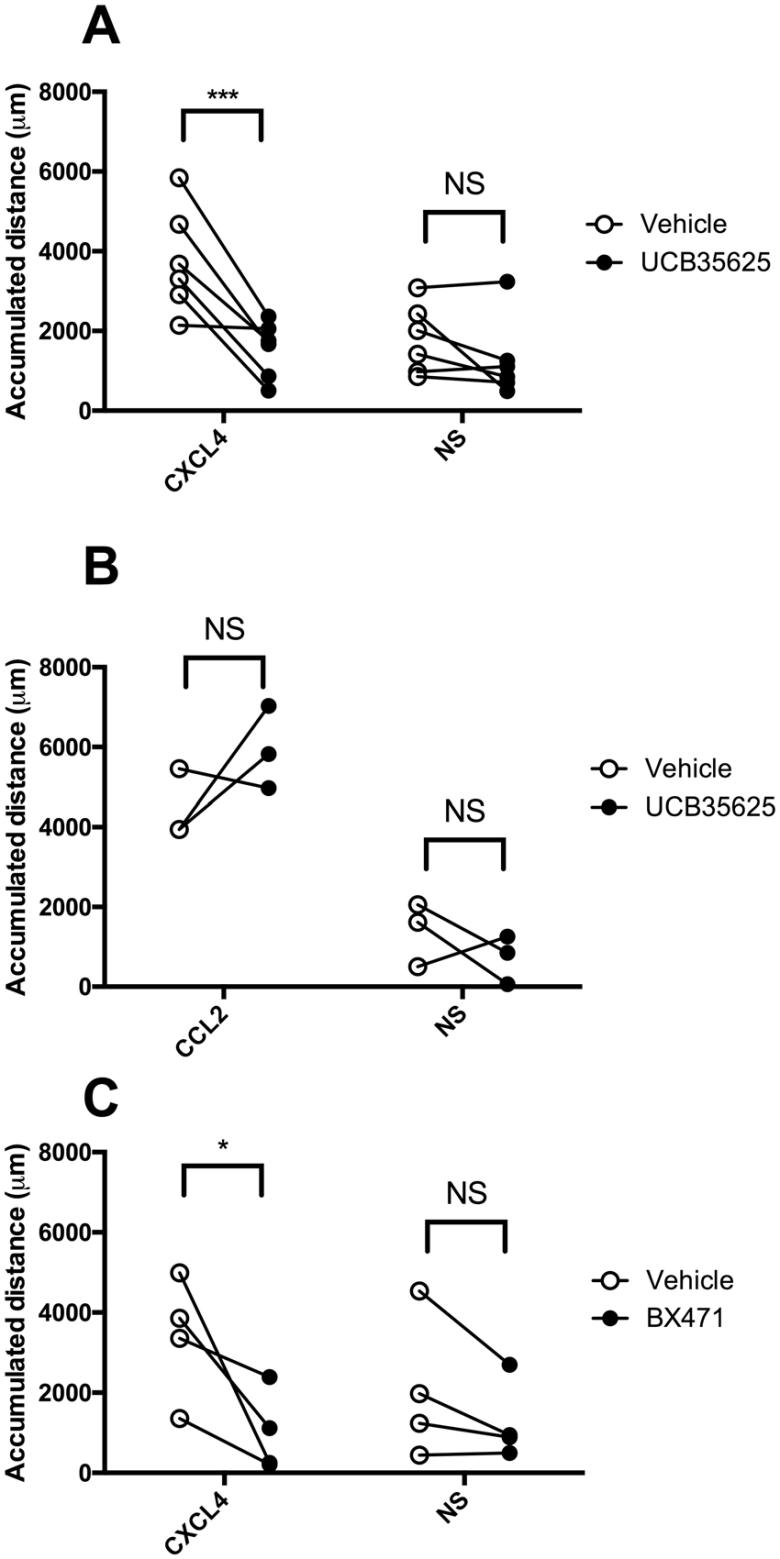
CXCL4-induced monocyte migration is sensitive to CCR1 antagonism- Panels A and B show the accumulated migration of monocytes in response to no stimulus, CXCL4 (Panel A, n = 6) or CCL2 (Panel B, n = 3) following pre-incubation with vehicle or the CCR1-antagonist UCB 35625. Panel C shows the accumulated migration of monocytes in response to no stimulus or CXCL4 following pre-incubation with vehicle or the CCR1-antagonist BX-471 (n = 4).

To confirm that the CXCL4-induced migration was mediated via CCR1, we assessed whether it was sensitive to pre-treatment with the CCR1 antagonists UCB 35625 and BX-471. The accumulated distance migrated by monocytes (i.e. the sum of the individual cell tracks) either along a CXCL4 gradient, along a CCL2 gradient or in the absence of stimulation was examined using monocytes pretreated with vehicle or with 10 *μ*M UCB 35625 or BX-471. Migration in response to CXCL4 was significantly inhibited by pretreated of cells with 10 *μ*M UCB 35625 when compared to vehicle treated cells (Fig. 10A) whilst basal migration, or migration along a gradient of CCL2 was unaffected (Fig. 10B). This is in line with our previous findings regarding the lack of activity of this compound in blocking CCL2 responses in monocytes^28^. Similarly, pretreatment of cells with 10 *μ*M BX-471, an alternative CCR1 antagonist, was seen to significantly inhibit the migratory responses to CXCL4 but not the basal migration (Fig. 10C).

## Discussion

The release of micromolar concentrations of CXCL4 by activated platelets has been implicated in the process of atherogenesis by virtue of its many effects upon monocytes, notably their endothelial arrest, recruitment to the sub-endothelial space and effects on survival and differentiation^10,14,20,23,31^. We present here a body of data that clearly identifies CXCL4 as a CCR1 agonist. Notably, we observed that CCR1 could induce the migration of CCR1-transfctants, albeit with reduced potency and efficacy when compared to the principal CCR1 ligand, CCL3. This is reminiscent of our earlier findings with CXCR3, where CXCL4 was observed to have reduced potency and efficacy when compared with the other CXCR3 ligands^17^. Since the current dogma suggests that chemokines are class restricted, i.e. CXC chemokines activate only CXC chemokine receptors, the finding that CXCL4 activates CCR1 was unexpected and serves to underline the apparent plasticity of some chemokine receptors with respect to ligand recognition. For example, *β*-defensin induces T-cell migration via CCR6^32^, whilst ubiquitin has been shown to be a ligand for CXCR4^33^. Similarly, the atypical chemokine receptor ACKR1, previously known as Duffy Antigen Receptor for Chemokines (DARC) is well known to bind chemokines of both CC and CXC classes^34^.

**Figure 11:**
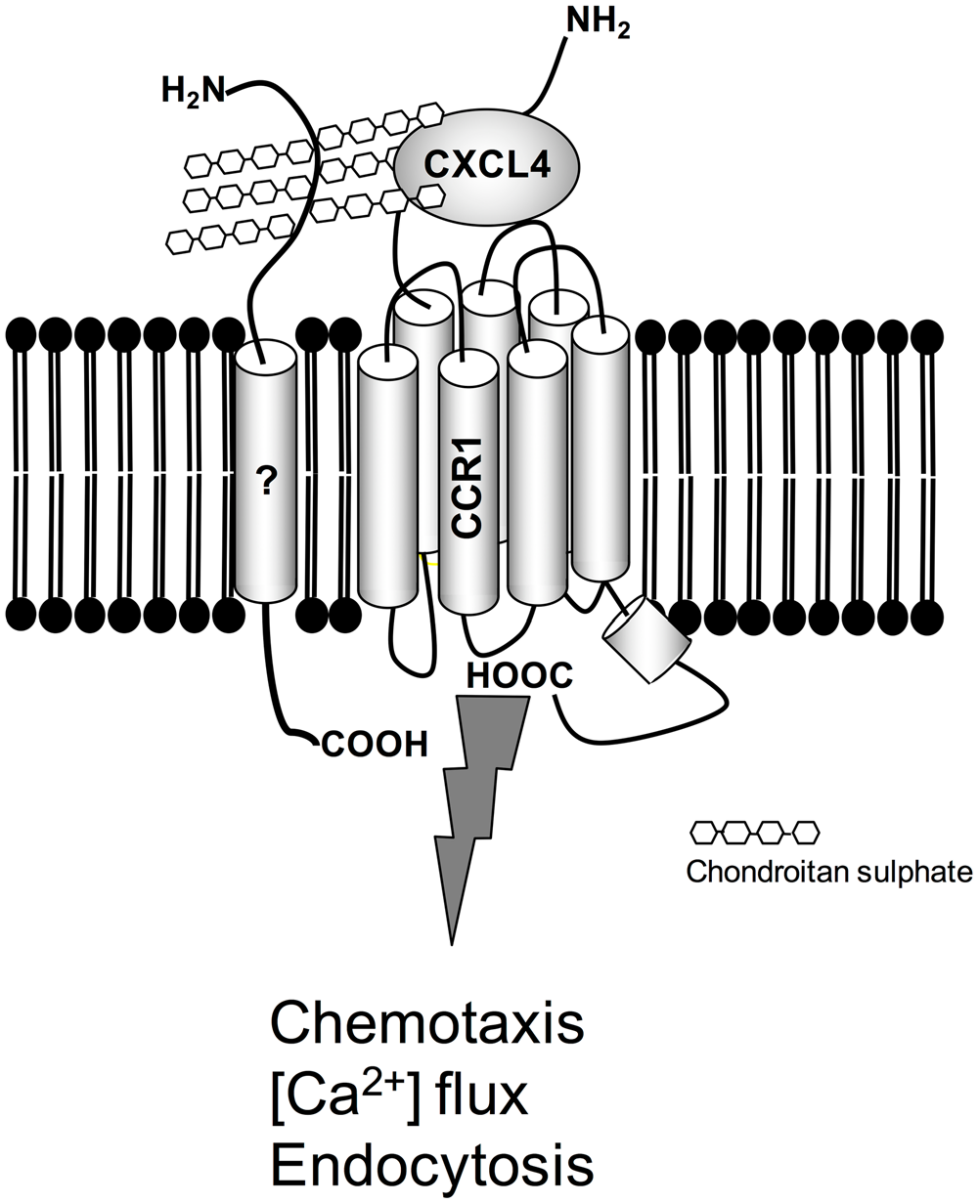
CXCL4 undergoes *cis* presentation to CCR1 to facilitate monocyte migration. The question mark denotes an as yet unidentified glycosaminoglycan decorated with chondroitin sulphate.

In keeping with the findings of many other groups studying aspects of CXCL4 signalling in a variety of cell types^12,17,18,22–24^, micromolar concentrations of CXCL4 were required to observe biological effects, suggesting that the affinity of CXCL4 for its receptor is likely to be low. Petersen and colleagues previously reported that CXCL4 bound to the surface of neutrophils with a *Kd* of less than 600 nM which they attributed to the binding of tetrameric CXCL4 to a chondroitin sulphate-containing cell surface glycoprotein of around 250 kDa, considerably larger than most GPCRs^25,26^. Similarly, von Hundelshausen and colleagues have shown that CXCL4-induced monocyte arrest on ECs requires chondroitin sulphate^14^. We observed that pre-treatment with the enzyme chondroitinase ABC markedly reduced the migration of THP-1 cells and CCR1 transfectants to CXCL4 which suggests that one or more glycoproteins decorated with chondroitin sulphate provide *cis* presentation of CXCL4 to CCR1 on the monocyte surface, which is critical for receptor activation (Fig. 11). A similar mechanism by which glycosaminoglycans on the surface of T-cells present CCL5 to the receptor CCR5 has previously been put forward^35^, with treatment with a cocktail of glycanases resulting in a loss of T-cell responses to CCL5. Consistent with this, we were unable to effectively compete CCL3 from the surface of CCR1 transfectants with CXCL4. This is reminiscent of our earlier study examining CXCL4 activation of CXCR3, where we were unable to demonstrate displacement of CXCL11 from CXCR3 by CXCL4, despite clearly showing CXCR3-mediated CXCL4 signalling, with glycosamingoglycans necessary for CXCL4 function^17^.

In addition to activity on CCR1 transfectants, we observed that CXCL4 could induce the migration of the monocytic cell line THP-1 in a modified Boyden chamber. These data were ably supported by realtime imaging of cell migration using a TAXIScan system in which CXCL4 clearly induced the directed migration of both THP-1 cells and monocytes. Although an earlier report suggested that CXCL4 was chemotactic for neutrophils and monocytes^13^, this was subsequently contested by Petersen and colleagues who failed to observe monocyte migration in response to a highly purified preparation of CXCL4 in Boyden chamber assays^12^. Their argument was that the original migration observed by Deuel and colleagues^13^ was likely due to contamination with other chemokines in the platelet-derived CXCL4 preparation. Here, we have used a highly pure recombinant form of CXCL4 (greater than 98 percent purity as determined by Western blotting and HPLC analysis) so can confidently attribute the observed chemotactic activity to CXCL4 alone. In keeping with our CCR1 transfectant data, CXCL4-induced migration of THP-1 cells and monocytes was CCR1-dependent, as shown by the action of the small molecule antagonists UCB 35625 and BX-471. It is unclear why in the Boyden chamber assays, the inclusion of chemokine above the membrane had no effect on CXCL4 responses, suggesting at face value, that the CXCL4-induced migration was chemokinetic (non-directional). This is clearly not the case, as the TAXIScan data (movies and VD-B plots) shows the migration to be chemotactic. The flaws of assessing chemokinesis in Boyden chambers are well known, hence the initial development of Dunn and Zigmond chambers and systems such as TAXIScan. It is debatable in modified Boyden chamber assays whether or not a chemoattractant gradient exists for the duration of the assay and the validity of the “checkerboard” correction for chemokinesis measurements has been questioned^36^. Our data highlights the need for direct visualisation to accurately examine the migratory behavior of cells in a chemotactic gradient, as suggested by others^37^.

In addition to its ability to induce monocyte arrest and migration, CXCL4 can also act as a differentiation and survival factor for monocytes, generating what has been dubbed the “M4” macrophage^23^. The transcriptional program induced by CXCL4 appears to be distinct from those induced by classical and non-classical activation, including apparently contradictory pro-atherosclerotic and anti-atherosclerotic components, such as down regulation of the atheroprotective scavenger CD163 and down regulation of the atherogenic scavenger CD36^20^. Notably, the M4 macrophage takes up much less modified low density lipoprotein (LDL) than those differentiated by M-CSF treatment^23^. If we extrapolate our findings here that CCR1 is a *bona fide* receptor for CXCL4 on monocytes, then our findings may explain at a mechanistic level why deletion of CCR1 in mice has been shown to enhance atherosclerosis in two independent studies^38,39^. This is in stark contrast to several other studies of chemokine receptor-deficient mice that display various levels of atheroprotection^4,5^. In light of our data here, we speculate that the accelerated atherosclerosis seen in the CCR1 deficient mice may be due to the selective loss of CCR1:CXCL4 signalling in macrophages within plaques that normally would lead to lower modified LDL uptake. Although CXCL4 deletion has previously been shown to be atheroprotective on the Apolipoprotein E null background^11^, such a broad stroke removes a number of potentially pro-atherosclerotic CXCL4 activities outside of CCR1 signalling, such as promoting oxidised LDL binding to the vasculature and inhibiting oxLDL endocytosis by the LDL receptor^40^ in addition to T cell recruitment via CXCR3-B^17^. We would suggest that when contemplating targeting CXCL4 in the treatment of atherosclerosis, alongside the pro-atherosclerotic activities of CXCL4, the anti-atherogenic properties of M4 macrophages must also be considered^41^.

Assuming the *in vitro* data described here extrapolates to the clinical setting, then it would strongly argue against targeting CCR1 in atherosclerosis and may point to unforeseen side effects following the long-term use of such molecules for the treatment of other inflammatory disorders. Of note, the small molecule CCR1 antagonist CCX354 has shown efficacy in a phase II clinical trial for the treatment of rheumatoid arthritis and larger, longer-term clinical trials have been planned^42^. Since accelerated atherosclerosis and increased mortality is often observed in arthritic patients (reviewed in^43^), blockade of CCR1 may be detrimental. Further experimentation to test such a hypothesis *in vivo* is highly desirable.

## Methods

### Materials

Recombinant chemokines were from PeproTech EC Ltd (London, UK). The CCR1 antagonists UCB 35625 and BX-471 were from BioTechne Ltd. (Abingdon, UK) and have previously been described^28,44^. *Bordetella pertussis* toxin and the mouse isotype-matched control IgG1 (MOPC 21 clone) were from Sigma-Aldrich (Poole, UK), as were heparinase and chondroitinase ABC. Radioiodinated CXCL10 and CCL3 were from Perkin Elmer Life Sciences (Boston, MA). The anti-human CCR1 and CXCR3 mAbs (MAB145 and MAB160, respectively) were from BioTechne Ltd. The anti-HA mAb was from Covance Biosciences (Crawley, UK). The secondary anti-mouse-FITC conjugated antibody was from DAKO UK Ltd, (Cambridge, UK). All other reagents, unless noted, were from Life Technologies (Paisley, UK).

### Cell Preparation and Maintenance

Blood was taken from healthy normal subjects with written informed consent. The protocol was approved by the Brompton, Harefield and NHLI ethics committee. All experiments were carried out in accordance with the approved guidelines. PBMCs were isolated as previously described^28^. THP-1 and L1.2 cells were maintained as previously described^44^. Where indicated, cells were treated overnight with 100 ng/ml *pertussis* toxin (Sigma-Aldrich) to inhibit G*α*i signaling. For studies of monocyte chemotaxis, monocytes were separated from whole blood using a Rosette-Sep monocyte purification kit (StemCell Technologies, Grenoble, France) according to the manufacturer’s instructions. Transient transfection of L1.2 cells with HA-tagged chemokine receptor constructs was by electroporation as previously described^44^.

### Flow cytometric analysis of chemokine receptor expression

Staining of HA-tagged CCR1, CCR2 and CCR3 transfectants was by the use of an anti-HA monoclonal as previously described^44^. Detection of CCR1 with MAB145 was as described previously^45^. Flow cytometry analysis was carried out using a LSR Fortessa or FACS Calibur (BD Bioscience, Oxford, UK); gating excluded cell debris and cell doublets from analysis.

### Cell Migration Assays

Modified Boyden Chamber assays were carried out as previously described^44^ using 5 *μ*m pore chambers (Neuroprobe Inc, Gaithersburg, MD). In experiments employing *pertussis* toxin, cells were pretreated with 100 ng/ml for 18 hours at 37 °C. Similarly, in experiments employing the glycosidases heparinase and chondroitinase ABC, cells were incubated with agitation with one unit/ml of enzyme for 30 minutes at 37 °C as previously described^25^, before being washed extensively in phosphate buffered saline. In experiments employing the CXCR3 or CCR1 antagonist, chemokine dilutions were made in a solution containing a fixed concentration of the competitor. In experiments to assess the contribution of chemokinesis, the same concentration of chemokine placed in the lower well of the chemotaxis chamber was present in the assay buffer, eliminating the chemoattractant gradient.

For real time analysis of migrating THP-1 cells and freshly isolated monocytes, a 12-channel TAXIScan was employed^27^ and used according to the manufacturer’s protocol (Effector Cell Institute, Tokyo. Japan). Where indicated, cells were pretreated with a fixed concentration of antagonist for 30 minutes at 37 °C prior to carrying out the assay. One *μ*l of a suspension containing 500,000 cells/ml was loaded into each chamber and following alignment of the cells at one end of the terrace, 1 *μ*l of chemokine (1 *μ*M CCL2 or 20 *μ*M CXCL4) was added to the other end of the terrace (260 *μ*m away) and cells were allowed to migrate along the ensuing chemokine gradient for 2 hr whilst maintained at 37 °C (Supplementary Figure 2). Sequential image data were captured at every minute as individual jpegs. To calculate the mean track length, data were subsequently processed with ImageJ (National Institutes of Health), equipped with the manual tracking tool plugin (Fabrice Cordelieres, Institut Curie, Orsay (France) and chemotaxis tool plugin (Ibidi, Martinsried, Germany). Individual experiments consisted of duplicate conditions and data illustrated are collated from several experiments as highlighted in the figure legend. The numbers of cells tracked under each condition are shown in the figure. For each individual cell, the mean track length was calculated by the chemotaxis tool plugin as described previously^46^. The accumulated distance parameter (Fig. 10) refers to the total distances traveled by all cells for a particular condition in an individual experiment.

Cell migration was also manually tracked using TAXIScan Analysis software in order to generate plots of Velocity versus Directionality. This is an accepted methodology for accurately distinguishing chemotaxis from chemokinesis with the TAXIScan system^27^. The direction of cell migration is expressed as the angle (rad) towards the concentration gradient, namely π/2 indicates that the cell is migrating toward the concentration gradient, whereas −π/2 indicates that the cell is migrating against the concentration gradient. Velocity-Directionality-Box (VD-B) plots were subsequently generated^47^ in which the vertical axis shows the median value of velocity and the horizontal axis shows the median value of the directionality. For THP-1 cells, tracking was undertaken during the initial 30 minutes of migration, whereas for slower moving monocytes, tracking was carried out for the first 60 minutes of migration.

### Intracellular Calcium Measurements

These were performed as previously described^19^ using cells that were loaded with the fluorescent dye FURA-2 AM (Life Technologies). Real time data were recovered using a fluorimeter (LS-50B, Perkin-Elmer, Beaconsfield, UK). Data are expressed as the relative ratio of fluorescence emitted at 510 nm after sequential stimulation at 340 and 380 nm.

### CCR1 Endocytosis Assays

THP-1 cells were cultured with 80 nM calcitriol (Sigma-Aldrich) for 48 hr to enhance CCR1 expression^48^. Similarly, CCR1-L1.2 transfectants were cultured overnight with 10 mM sodium Butyrate to enhance CCR1 expression^44^. Human monocytes were used within an hour of purification. Cells were resuspended at 500,000 cells/ml in RPMI, 0.1 % BSA and kept on ice. Aliquots of these cells (50 *μ*l) were incubated with either an identical volume of buffer alone or buffer containing CCL3 or CXCL4, giving a final concentration of 50 nM CCL3 or 1 *μ*M CXCL4. Samples were either left at 4 °C for 30 minutes or incubated at 37 °C for 30 minutes after which they were stained for CCR1 expression by flow cytometry as described above. Data is presented as the percentage of CCR1 staining observed in the buffer treated samples that were incubated on ice.

### Statistical Analysis

Statistical analyses were carried out using Prism 6 (GraphPad Software, La Jolla, CA). Unless otherwise stated, data reflect the mean values ± SEM from the number of experiments shown in parenthesis. Unless otherwise stated, statistics refer to repeated measures ANOVA with Bonferroni’s post-test. *P ≤ 0.05, **P ≤ 0.01, ***P ≤ 0.001 and ****P ≤ 0.0001. NS denotes not significant.

### Data Availability

The datasets generated during and/or analysed during the current study are available from the corresponding author on reasonable request.

## Electronic supplementary material

Supplementary Information

Supplementary BBL

Supplementary Video 1

Supplementary Video 2

Supplementary Video 3

Supplementary Video 4

Supplementary Video 5

Supplementary Video 6
