# Supplementary material for: CXCL4/Platelet Factor 4 is an agonist of CCR1 and drives human monocyte migration
Source: Sci Rep. 2018 Jun 21;8:9466. doi: 10.1038/s41598-018-27710-9 (PMC6013489; doi:10.1038/s41598-018-27710-9)
Supplement: Supplementary file 1 — Supplementary Information [file 41598_2018_27710_MOESM1_ESM.pdf]

# CXCL4/Platelet Factor 4 is an agonist of CCR1 and drives human monocyte migration

James M. Fox, Fahima Kausar, Amy Day, Michael Osborne, Khansa Hussain, Anja Mueller, Jessica Lin, Tomoko Tsuchiya, Shiro Kanegasaki, and James E. Pease

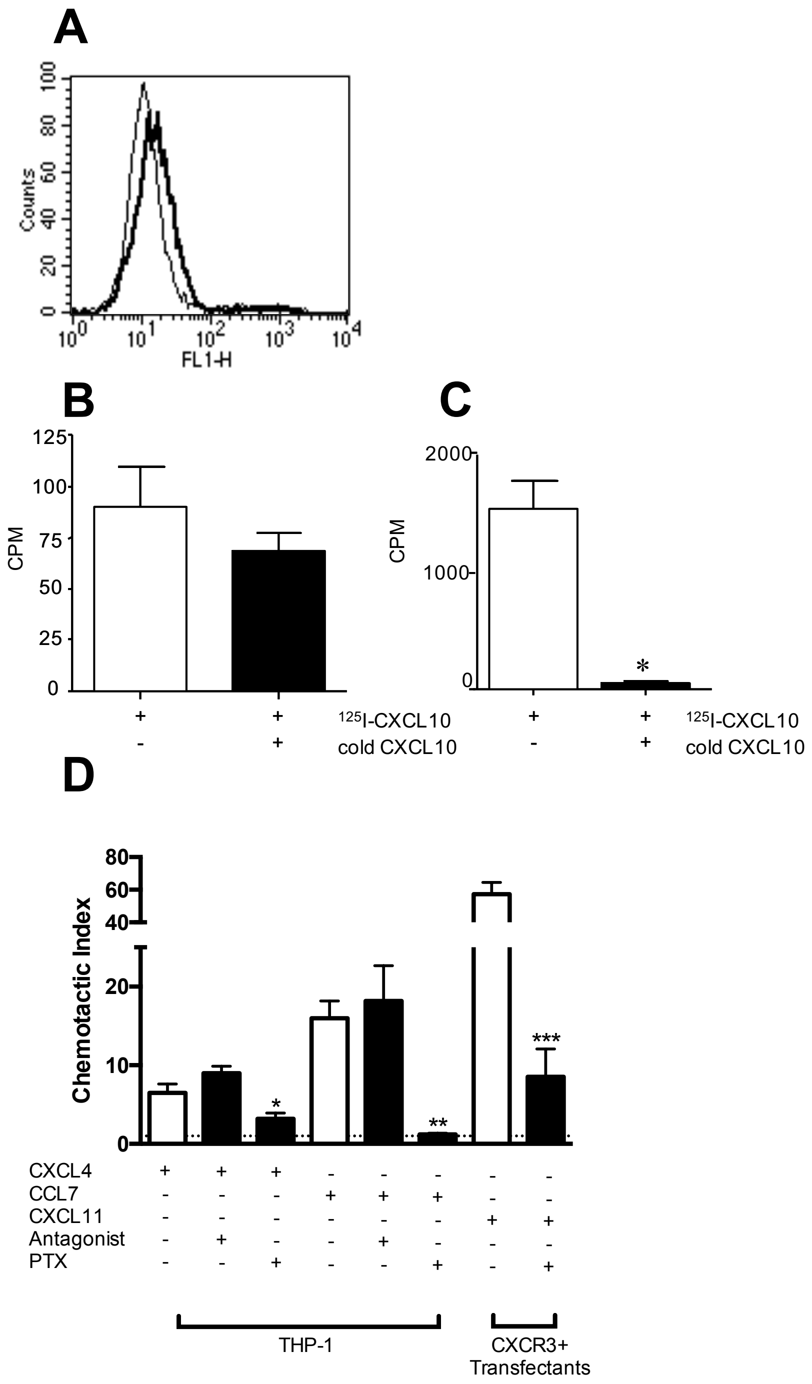


**Supplementary Figure 1. CXCL4 interacts with a receptor on THP-1 cells distinct from CXCR3**

Panel A. Representative image showing low levels of cell surface CXCR3 as deduced from flow cytometric analysis of THP-1 cells with either an anti-CXCR3 antibody (bold line) or an isotype control antibody (faint line).

Panel B. Lack of specific binding of $^{125}$I-CXCL10 to THP-1 cells, showing the counts per minute (CPM) obtained when 0.1 nM $^{125}$I-CXCL10 was allowed to bind to THP-1 cells in the presence of buffer alone or 300 nM of unlabelled CXCL10. Panel C. Binding of 0.1 nM $^{125}$I-CXCL1 0 to activated T-lymphocytes in the presence of buffer alone or 300 nM of unlabelled CXCL10. Panel D. Migratory responses of THP-1 cells or CXCR3 transfectants to 10 nM CCL7, 1 µM CXCL4 or 50 nM CXCL11 following pre-treatment of the cells with *pertussis* toxin or in the presence or absence of a specific CXCR3 antagonist (2 µM). Dotted line depicts basal migration at chemotactic index = 1. * Indicates significant difference ($P<0.05$) compared with the untreated control by way of unpaired t-test. In panels B-D, data shown are the mean ± SEM of 3 experiments.

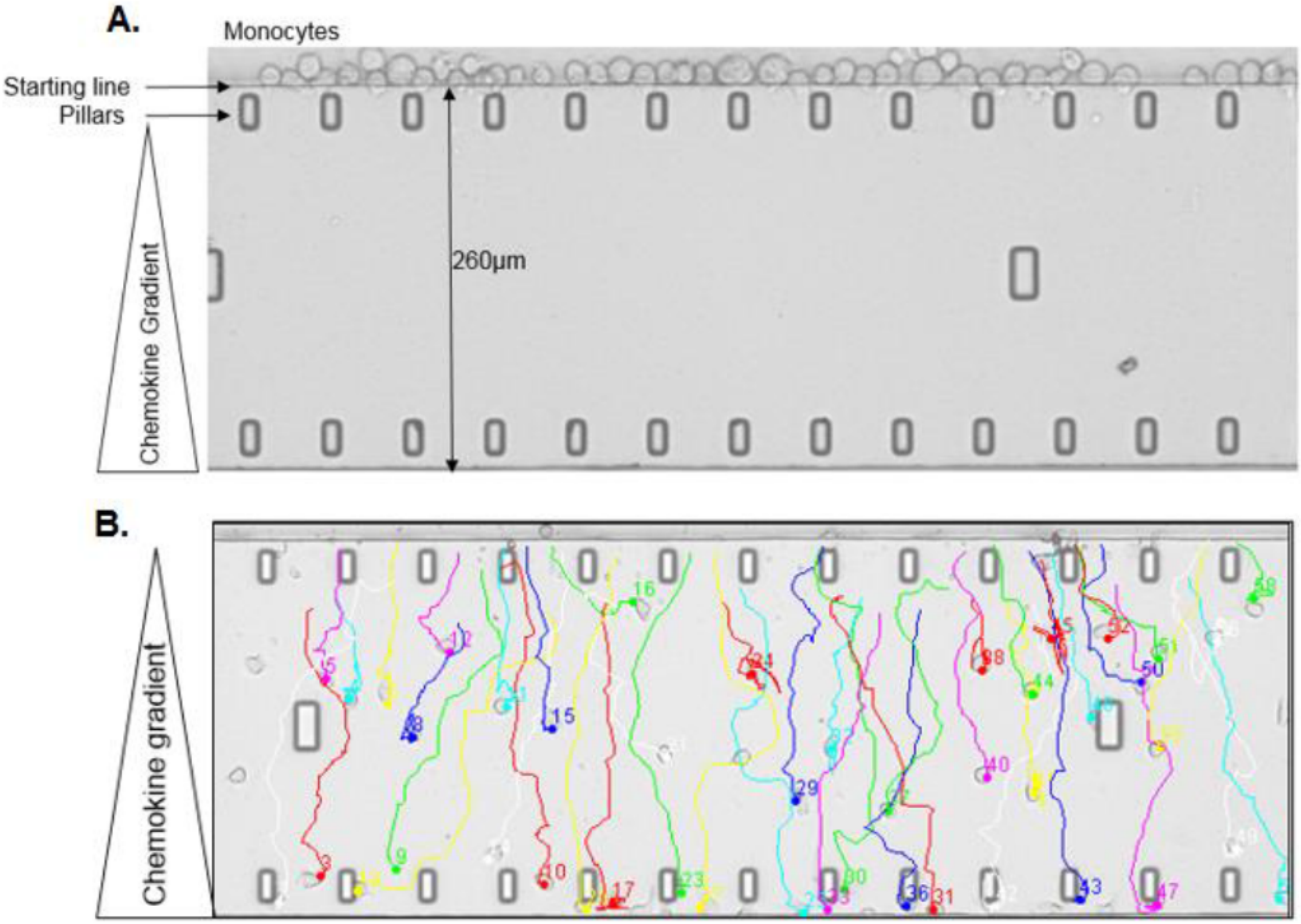


**Supplementary Figure 2 - Analysis of cell migration via TAXIScan**

Panel A shows monocytic THP-1 cells aligned at the uppermost surface of the field of view prior to migration.

Panel B shows migration of the monocytes in the same chamber after 2hrs of exposure to a concentration gradient formed by the addition of CCL2 to the opposite end of the terrace (diameter 260 µm). To migrate along the gradient, the cells must squeeze between the celling and floor of the terrace (height 5 µm). Images were taken every 60s and cells were manually tracked in Image J. The individual cell tracks are shown in colour and are labelled. Only cells that migrated onto the terrace past the indicated pillars were imaged.

**Supplementary Videos 1, 2 & 3- THP-1 migration observed via TAXIScan**

Migration of THP-1 cells along a CCL2 gradient (Supplementary Video 1), along a CXCL4 gradient (Supplementary Video 2) or in the absence of stimulation (Supplementary Video 3) detected using the TAXIScan device. Elapsed time in minutes and seconds is indicated in the bottom right hand corner. Scale bars indicate 100 microns.

**Supplementary Videos 4, 5, & 6 - Human monocyte migration observed via TAXIScan**

Migration of freshly isolated human monocytes along a CCL2 gradient (Supplementary Video 4), along a CXCL4 gradient (Supplementary Video 5) or in the absence of stimulation (Supplementary Video 6) detected using the TAXIScan device. Elapsed time in minutes and seconds is indicated in the bottom right hand corner. Scale bars indicate 100 microns.
